# roadDB: An R package for exploring early human prehistory and environments with the ROCEEH Out of Africa Database

**DOI:** 10.1371/journal.pone.0359952

**Published:** 2026-10-07

**Authors:** Christian Sommer, Jesper Borre Pedersen, Zara Kanaeva, Timo P. Streicher, Andrew W. Kandel, Nicholas J. Conard, Volker Hochschild

**Affiliations:** 1 The Role of Culture in Early Expansions of Humans, Heidelberg Academy of Sciences and Humanities, Tübingen, Germany; 2 Department of Geosciences, Institute of Geography, University of Tübingen, Tübingen, Germany; 3 Department of Geosciences, Working Group Early Prehistory and Quaternary Ecology, University of Tübingen, Tübingen, Germany; University of Florida, UNITED STATES OF AMERICA

## Abstract

Macroscale and interdisciplinary approaches are emerging as central paradigms in the study of human evolution, as demonstrated by a growing body of large-scale, data-driven research. This development is paralleled by an expanding ecosystem of R packages that provide access to paleoenvironmental and paleoclimatic reconstructions, as well as advanced tools for spatial and temporal analysis and modeling. However, within this computational framework, direct programmatic access to comprehensive archaeological and anthropological data remains limited. We present roadDB, an R package that enables streamlined access to the ROCEEH Out of Africa Database (ROAD), a major repository of archaeological, paleoanthropological, paleoenvironmental, geographical and chronological data relevant to human evolution. roadDB bridges the gap between the existing R ecosystem and the ROAD archaeological dataset that covers most of the last 3 million years of human evolution across Africa and Eurasia and thereby represents the only openly available archaeological dataset with such an extensive temporal and geographic coverage. In short, roadDB offers the research community unrestricted, transparent access to a richly curated collection of site-level and assemblage-level information, opening new possibilities for large-scale quantitative research. The package strengthens the integration of archaeological and paleoenvironmental evidence in macroscale analyses and supports robust, transparent and interdisciplinary research on the spatio-temporal dynamics of human evolution.

## 1. Introduction

Large-scale, integrative databases are increasingly central to research on human evolution and deep prehistory [1]. Kintigh and colleagues [2] proclaimed, that the grand challenges in archaeology require systematically collected data, modeling and synthetic research. Addressing questions that span hundreds of millennia and entire continents requires data infrastructures that combine archaeological, paleoanthropological, paleoenvironmental, and chronological evidence within a unified spatio-temporal framework. The ROCEEH Out of Africa Database (ROAD [3]) was developed precisely to meet this challenge, providing a curated, relational repository of information on the deep human past across Africa and Eurasia between 3,000,000 and 20,000 years ago. This resource has been widely used, especially in recent years (see https://www.zotero.org/groups/5497463/roceeh/library). While ROAD offers a powerful web-based interface for data exploration and manual downloads, many contemporary research questions require programmatic, reproducible, and extensible workflows. Increasingly, archaeological inference relies on computational pipelines that integrate multiple data sources, apply transparent transformations, and enable replication and reuse [4,5]. In this context, the lack of a native scripting interface to ROAD has represented a significant bottleneck. The roadDB R package was developed to bridge this gap. It provides structured, script-based access to ROAD data directly within R [6], allowing researchers to integrate ROAD seamlessly into reproducible analytical workflows. In doing so, roadDB lowers barriers to accessing the ROAD database; it paves the way for multi-scalar and interdisciplinary research about the early human past based on transparent, reproducible analytical workflows.

### 1.1 Human evolutionary studies at the macro scale

In recent years, there has been a marked increase in scholarly contributions addressing human evolution and paleoenvironmental dynamics across extended spatial and temporal scales. This paradigm shift is driven by two mutually reinforcing factors. These include: 1) technological advances that have rendered computational methods increasingly cost-efficient as well as 2) a broader transformation toward open and FAIR (Findable, Accessible, Interoperable, Reusable) science [7]. Within this evolving research culture, data sharing has become not only standard practice but also an expected norm of good scientific conduct [8], paralleled by the growing institutionalization of code sharing as a core component of transparent and reproducible research [9].

These developments widened the scope of prehistoric research in terms of demography [10–13] and the evolution of material culture over hundreds of millennia and continental scales [14–17]. The growing reach of paleoenvironmental reconstruction allows the study of the early associations between cultural expression and environments [18–22], environmental resources [23] or the interaction of early humans with other taxa [24]. A variety of ecological niche models has been applied to estimate the potential distribution of diverse human species or cultures [25–43] and the niches of faunal taxa [44,45]. By combining paleoanthropological datasets, one can study the drivers of macroscale biological evolution of hominin species [46]. Geographical expansions are modeled by means of least cost path (LCP) and agent-based modeling (ABM) [47–50]. Increasingly, machine learning (ML) and artificial intelligence (AI) permeate all fields related to the quantitative study of human prehistory [51].

These quantitative and computer-driven examples reflect the emergence of data-centric prehistoric research [52]. The scripting language R plays a central role in this development [53], having developed into a comprehensive ecosystem for human evolutionary research through an extensive array of domain-specific packages and reproducible workflows. It is therefore only logical to introduce roadDB, integrating the most comprehensive database of its kind, into this ecosystem as a native data source.

### 1.2 Background to the ROCEEH Out of Africa Database (ROAD)

roadDB is an R package that provides simplified programmatic access to ROAD, a curated interdisciplinary database developed since 2008 by the research center “The Role of Culture in Early Expansions of Humans” (ROCEEH), funded by the Heidelberg Academy of Sciences and Humanities and located at the University of Tübingen, Germany, and the Senckenberg Research Center, Frankfurt, Germany. Unique in its spatial and temporal scope it currently encompasses more than 29,000 assemblages from over 2,700 localities, derived from more than 6,500 scientific publications in over 10 languages.

ROAD is a geospatial data infrastructure designed to integrate disciplinary information from archaeology (material culture and traces of human modification), anthropology (human fossil remains), paleontology (faunal remains), and paleoenvironmental archives (botanical remains). Much of this information is structured at the assemblage level. Interdisciplinary integration is achieved by situating assemblages within localities and their stratigraphic context, implemented as geological and archaeological layers. Chronometric information derived from these layers, as well as from individual finds, extends the geospatial framework with a temporal dimension. Publications are linked to localities, ensuring that the provenance of stored information can be transparently traced back to its original sources.

At the core of this infrastructure lies a PostgreSQL relational database comprising 68 tables and numerous attributes. This structure reflects the high degree of detail and formal organization of the disciplinary data. At the same time, it underscores ROAD’s status as an expert database, requiring both technical skills to formulate meaningful queries and domain-specific knowledge to interpret its contents.

To facilitate access for different user groups, several user interfaces have been developed:

- ROAD Simple Search provides a dashboard offering a condensed overview of sites and broad find categories via an interactive web map and filterable table.
- Summary Data Sheets present site-specific information in reader-friendly, graphically structured PDF format.
- AskROAD enables customized downloads of predefined data tables in open formats such as CSV, JSON, and XML through a guided web interface.
- The ROAD Query Tool (registration required) allows users to formulate interactive SQL queries in flexible ways, supporting complex spatio-temporal analyses. Results can be downloaded as csv files or visualized in the Map Module, a WebGIS viewer.
- The SPARQL Endpoint provides semantic web access via the SPARQL Protocol and RDF Query Language, enabling interoperability with linked data environments.

Among these interfaces, the ROAD Query Tool and AskROAD provide downloadable, machine-readable data in open formats. However, practical experience has shown that the ROAD Query Tool can be overwhelming for many users, as it requires technically correct SQL statements and a comprehensive understanding of the database structure. AskROAD reduces these technical barriers by offering predefined query templates, though its limit is quickly reached with complex multidisciplinary queries. It also requires subsequent formatting and data cleaning for integration into analytical software.

These limitations highlight the need for direct programmatic integration within analytical environments. Such integration enhances reproducibility, eases interoperability with other analytical tools, and lowers barriers for large-scale or iterative analyses.

## 2. Methods

The roadDB package provides wrapper functions that establish a connection to the online ROAD database, construct and execute structured query language (SQL) statements, and return query results in a standardized data frame format suitable for downstream analysis. A structural overview is presented in Fig 1.

**Fig 1:**
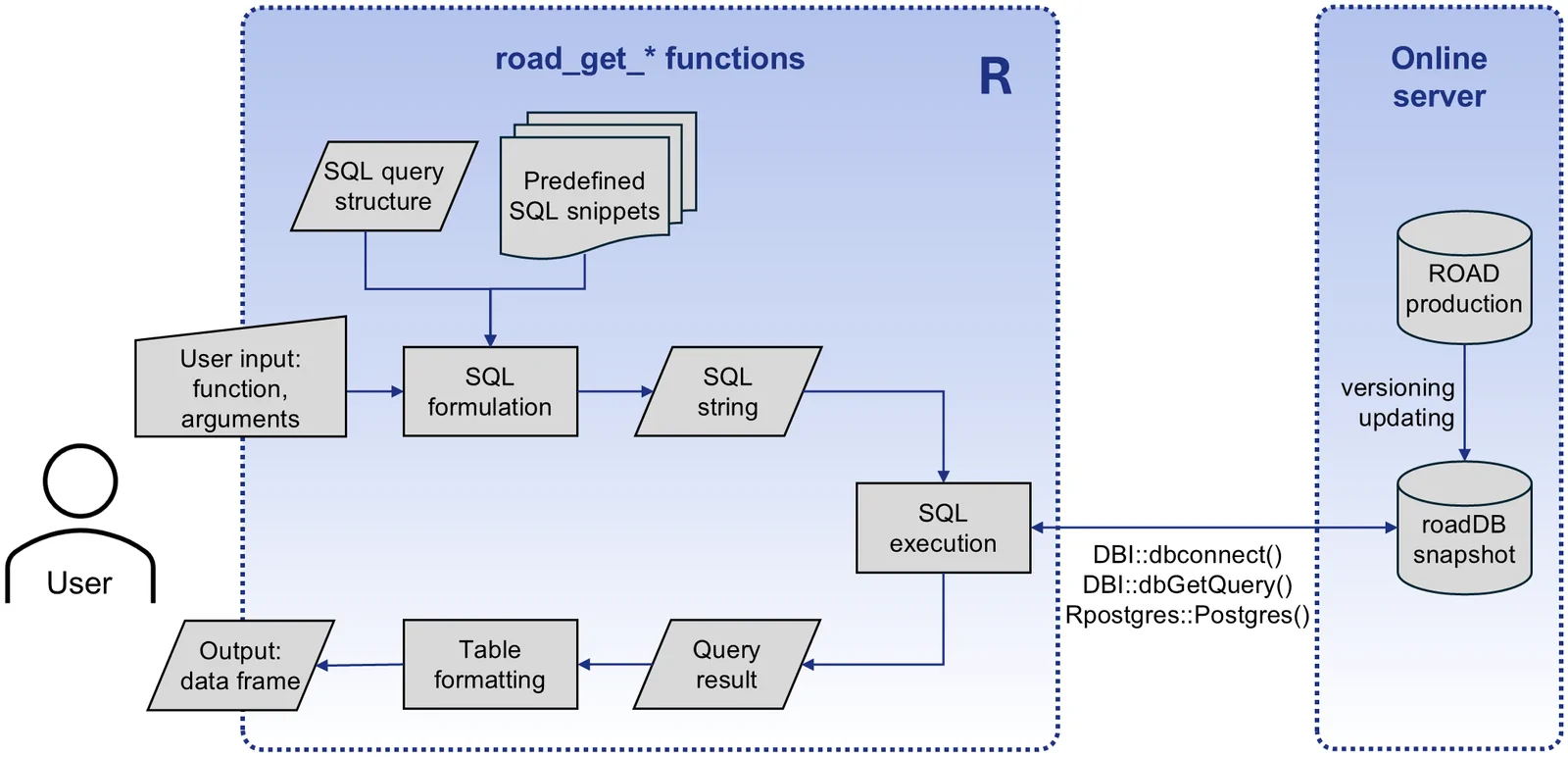
Workflow and system architecture. In a typical road_get_* function, the user provides inputs using standard R syntax. User-supplied information (such as function type, arguments, and values) is internally combined with a basic SQL query structure and predefined SQL snippets. This creates a custom SQL string, which is then queried against the online roadDB database. The roadDB database is a snapshot of the ROAD production system and updated regularly every three months. The query result received from the database are formatted as standard data frames and returned to the user. Note that query results may differ each time the roadDB database is updated.

### 2.1 Online database architecture

The architectural backbone of the package is the roadDB PostgreSQL database called “snapshot”, which represents a regularly updated, exact replica of ROAD’s primary production system. While the main production database is continuously modified and extended, the snapshot is derived from it at quarterly intervals.

This structural separation serves multiple purposes. First, it enhances security by preventing direct access to the production system and mitigating risks such as SQL injection and unauthorized data manipulation. Access via the roadDB package is restricted through read-only permissions.

Second, the periodically updated snapshot can be versioned systematically, unlike the continuously evolving production system. This versioning ensures that identical queries yield consistent results, as long as the snapshot is active. The snapshot is updated every three months. Consequently, the approach supports reproducibility of analyses and enhances transparency in scientific reporting, e.g., by stating the snapshot version date. Upon loading the package, users are provided with a URL that provides information about the current snapshot version and online status (https://github.com/sommergeo/roadDB?tab=readme-ov-file#roaddb--).

### 2.2 Data retrieval workflow

The roadDB functions foster interoperability and reusability by replacing the manual formulation of SQL queries, which many users report as a significant barrier. The road_get_* functions assemble SQL statements following the general structure shown below. The specific expressions are determined by the function selected and the arguments supplied by the user.

Depending on the function type, the result fields (columns) are specified using the SELECT clause, while the required tables and joins are defined via the FROM clause. Predefined SQL code snippets are dynamically assembled to ensure that all necessary tables are correctly linked. User-supplied arguments are incorporated through the WHERE clause, with conditions and filter predicates constructed dynamically from predefined SQL snippets and the supplied argument values. The function’s level of detail (see section 3.2) determines how rows are aggregated using the GROUP BY clause. Results are ordered alphabetically by locality name.

SELECT

   Fields expression

FROM

   Table identifiers and joins

WHERE

   Condition and filter predicates

GROUP BY

   Predefined aggregation expression

ORDER BY

   Predefined order expression

The functions then connect internally to the database snapshot and execute the SQL statement using functions of the R database interface package ‘DBI’ (v.1.3.0 [54]) together with the ‘RPostgres’ driver (v.1.4.10 [55]). The query results are returned as formatted data tables via function output.

### 2.3 Open licensing

This package is released under the Creative Commons Attribution–ShareAlike 4.0 International (CC BY-SA 4.0) license. Although this license was not specifically designed for software, it ensures full compatibility with the original ROAD dataset, which is distributed under the same terms. Using CC BY-SA 4.0 maintains legal consistency between the source data and the package, allowing redistribution and adaptation under identical conditions while preserving attribution and share-alike requirements.

## 3. Description

### 3.1 Package overview

The R package roadDB is available on CRAN under https://doi.org/10.32614/cran.package.roaddb and the development version is on GitHub at https://github.com/sommergeo/roadDB. It allows users to obtain ROAD data in R through a clear and consistently named set of retrieval functions: the road_get_* functions (Fig 2). These functions provide optional and flexible filtering arguments and return results as clean data frames that follow the tidy data schema [56]. In this format, each variable forms a column, each observation forms a row, and each value occupies a single cell. This design improves interoperability with the wider R ecosystem, particularly the ‘tidyverse’, whose packages share common data structures and a consistent grammar for data import, transformation, and visualization [57]. In practice, this means that roadDB outputs can be used immediately with auxiliary tools (see section 4.1) without manual cleaning and with minimal reshaping. The retrieval functions are complemented by helper functions that support users in applying them effectively and conveniently.

**Fig 2:**
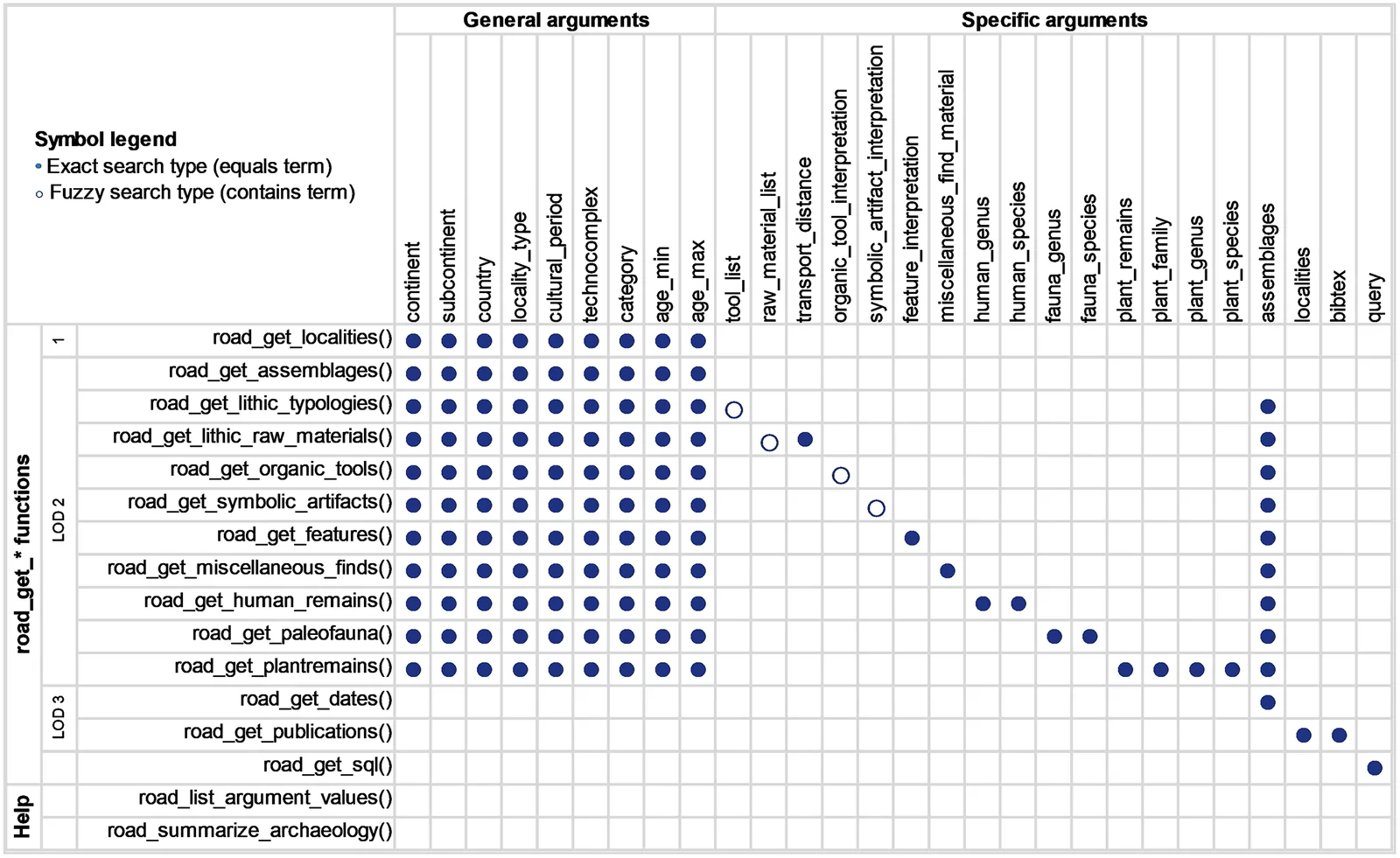
Compact overview of the available combinations of functions and arguments. The cross table summarizes all possible combinations of functions and arguments. The functions are grouped into three levels of detail (1 = site, 2 = assemblage, 3 = object) as well as helper functions. General arguments can be used with all functions at the site and assemblage level. Specific arguments are dedicated to individual functions. Filled dots indicate that the argument accepts exact values (e.g., continent = “Africa”), whereas hollow dots indicate that arguments accept fuzzy values (e.g., tool_list=”blade” matches “blades” and “bladelets”).

### 3.2 road_get_* functions

The road_get_* functions (Table 1) define the observational unit (rows) and associated variables (columns) of the returned tables. A key design feature of roadDB is that these retrieval functions follow three Levels of Detail (LOD), which determine the granularity of the output:

- LOD 1 (Locality-level): Each row represents a locality (site), including attributes such as geographic coordinates, locality type, cultural attributions, and ROAD-derived age ranges.
- LOD 2 (Assemblage-level): Each row represents an assemblage, including details on contents such as lithic typology, symbolic artifacts, organic tools, or human, faunal, and botanical remains.
- LOD 3 (Object-level): Each row represents an individual object, such as chronometric dates and publication sources.

**Table 1:** Summary of all retrieval functions, their level of detail and a short description of contents.

Function	Level of Detail	Description
road_get_localities()	1 – Locality	Geographic, cultural, and temporal data for sites
road_get_assemblages()	2 – Assemblage	Assemblage-level summaries
road_get_lithic_typologies()	2 – Assemblage	Lithic tool types and typological attributes
road_get_lithic_raw_materials()	2 – Assemblage	Lithic raw material categories and transport information
road_get_symbolic_artifacts()	2 – Assemblage	Symbolic and ornamental artifacts
road_get_organic_tools()	2 – Assemblage	Bone, antler, ivory, and other organic tools
road_get_features()	2 – Assemblage	Archaeological features (e.g., hearths, pits, constructions)
road_get_miscellaneous_finds()	2 – Assemblage	Non-standard or residual artifact categories
road_get_human_remains()	2 – Assemblage	Human fossils and taxonomic attribution
road_get_paleofauna()	2 – Assemblage	Faunal remains and taxonomic attribution
road_get_plantremains()	2 – Assemblage	Botanical remains and taxonomic attribution
road_get_dates()	3 – Object	Individual absolute dates (e.g., radiocarbon, U-series, etc.)
‍‍‍road_get_publications()	3 – Object	Individual source publications
road_get_sql()	–	Customized requests in Structured Query Language give experts full read access to the whole database

This tiered structure enables users to move from macro-scale questions (e.g., mapping technocomplex distributions) to fine-grained chronological or assemblage-specific analyses, while keeping outputs consistent and joinable across analytical steps.

The arguments determine which subset of the data is retrieved by applying filters based on geography, time, cultural attribution, or content. Arguments are optional. If no arguments are supplied, a road_get_* function returns all available records of the corresponding domain from ROAD. In practice, however, most research questions require more targeted datasets. Queries can therefore be refined by specifying one or more arguments, for example, to restrict results to a particular continent or cultural period. Arguments can be combined freely, and users may supply either single values or vectors of values. For example:

# Single argument

road_get_localities(cultural_period = "MSA")

# Multiple arguments

road_get_localities(cultural_period = "MSA",

            country = "South Africa")

# Lists of values

road_get_localities(cultural_period = c("MSA", "LSA"),

            country = c("South Africa", "Namibia"))

The package distinguishes between two classes of arguments (Table 2). First, general arguments are available across all road_get_* functions and provide flexible, consistent filters for spatial, temporal, and cultural dimensions of the data. Second, function-specific arguments are implemented only for selected functions and enable refinements unique to particular domains (e.g., lithic typology, dating methods, or biological taxonomy). Some of these arguments require exact matches to standardized ROAD terminology, whereas others support fuzzy matching to facilitate exploratory queries (Fig 2).

**Table 2:** Summary of general and function-specific arguments, their respective functions, values, match types, and examples. Character values are always entered within quotation marks.

Argument	Applies to function	Argument type	‍Value type	Match type	Example
continent	All road_get_* functions	general	character‍	exact	“Africa”
subcontinent	All road_get_* functions	general	character	exact	“Southern Africa”
country	All road_get_* functions	general	character	exact	“South Africa”
locality_type	All road_get_* functions	general	character	exact	“rock shelter”
cultural_period	All road_get_* functions	general	character	exact	“Middle Paleolithic”
technocomplex	All road_get_* functions	general	character	exact	“MSA/ Still Bay”
category	All road_get_* functions	general	character	exact	“symbolic artifacts”
age_min	All road_get_* functions	general	numeric	exact	20000
age_max	All road_get_* functions	general	numeric	exact	300000
tool_list	road_get_lithic_typologies()	general	character‍	fuzzy	“levallois”
raw_material_list	road_get_lithic_raw_materials()	specific	‍character	fuzzy	“ironstone”
transport_distance	road_get_lithic_raw_materials()	specific	‍character	exact	“local (0-5 km)”
organic_tool_interpretation	road_get_organic_tools	specific	character	fuzzy	“point”
symbolic_artifact_interpretation	road_get_symbolic_artifacts()	specific	character	fuzzy	“ornament”
feature_interpretation	road_get_features()	specific	character	exact	“hearth”
miscellaneous_find_material	road_get_miscellaneous_finds()	specific	character	exact	“mineral pigment”
human_genus	road_get_human_remains()	specific	character‍	exact	“Homo”
human_species	road_get_human_remains()	specific	‍character	exact	“sapiens”
fauna_genus	road_get_paleofauna()	specific	character‍	exact	“Equus”
fauna_species‍	road_get_paleofauna()	specific	character	exact	“capensis”
‍‍‍‍plant_remains	road_get_plantremains()	specific	character	exact	“pollen”
plant_family	road_get_plantremains()	specific	‍character	exact	“Fagaceae”
‍‍‍‍plant_genus	road_get_plantremains()	specific	‍character	exact	“Quercus”
‍‍‍‍plant_species	road_get_plantremains()	specific	‍character	exact	“ilex”
‍‍‍‍assemblages	road_get_dates()	specific	dataframe		Results from LOD 2 functions
‍‍‍‍localities	road_get_publications()	specific	dataframe		Results from LOD 1 functions
query	road_get_sql()	specific	character	exact	Custom SQL string

### 3.3 Helper functions

Finally, roadDB includes a set of helper functions (Table 3). All road_get_* functions are accompanied by detailed documentation accessible through R’s standard *help()* system. This documentation specifies the purpose of each function, the arguments it accepts, the structure and meaning of the returned columns, and guidance on how the output should be interpreted.

**Table 3:** Summary of helper functions and their purpose in roadDB.

Function	Argument	Purpose
help()	‍function name	Detailed documentation for each function
road_list_argument_values()	‍argument name	List valid values for a given argument
road_summarize_archaeology()	‍custom search term	Identify where a term occurs across archaeological tables

To support accurate and reproducible querying, roadDB provides the helper function *road_list_argument_values()*, which returns all valid values associated with a given argument. This is particularly useful when working with standardized archaeological terminology in ROAD, such as technocomplex names or taxonomic classifications, where exact spellings are required. By displaying the set of permissible values, this function enables users to explore available options before constructing queries.

Archaeological entities and materials may occur across multiple ROAD tables and attributes. The function *road_summarize_archaeology()* facilitates cross-table discovery by summarizing where a given search term appears within the archaeological datasets. The resulting summary table indicates which road_get_* functions contain the term and identifies the relevant columns. This allows users to target subsequent queries more efficiently and avoid unnecessary data retrieval.

### 3.4 Examples

The following examples demonstrate how data obtained through roadDB can be easily integrated in external packages and existing workflows. The reproducible R code to all examples in Fig 3 can be found in the S1 File. Further examples and more complex analyses can be found in our dedicated tutorial (https://www.sommergeo.com/roadDB/tutorial.html).

**Fig 3:**
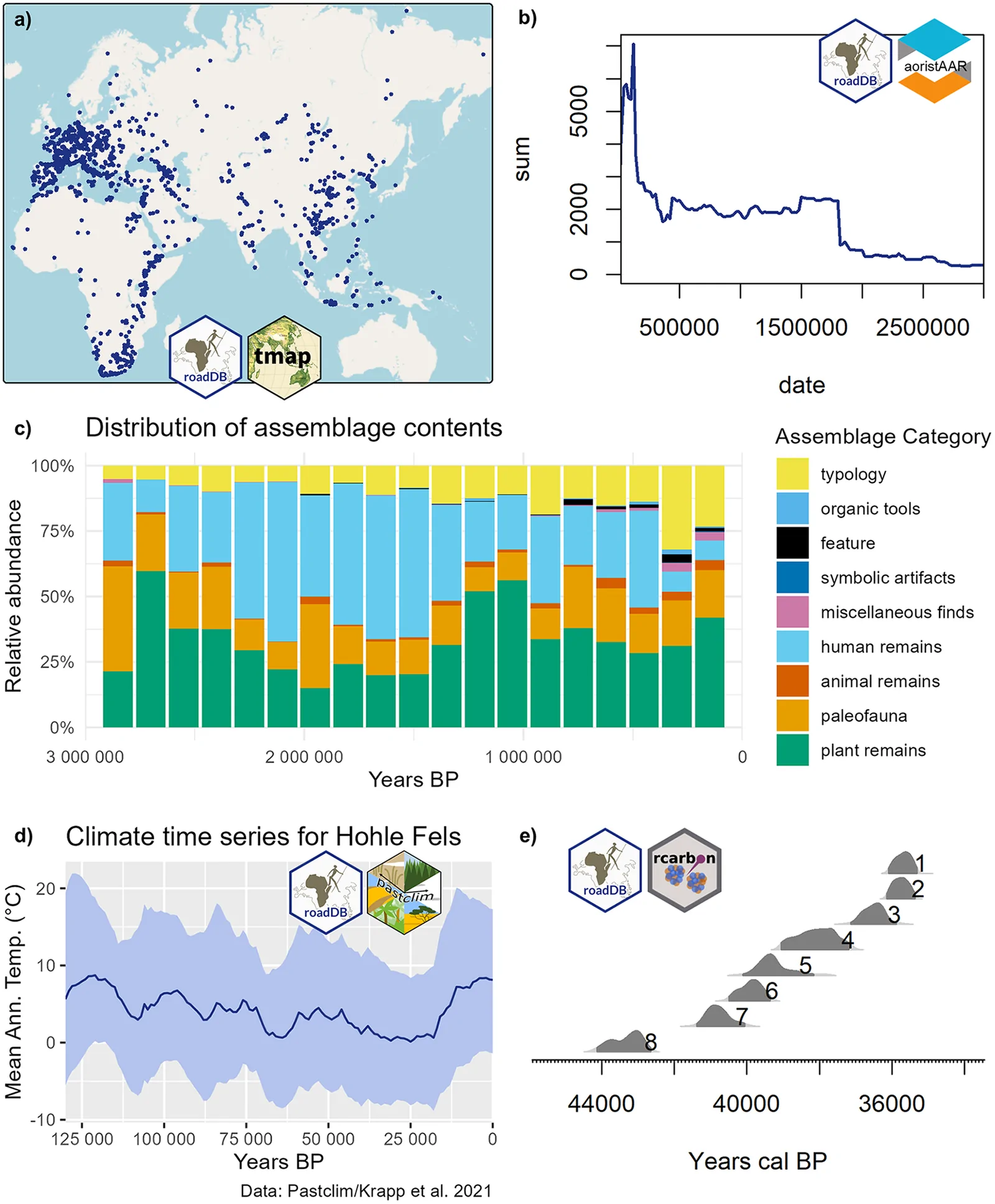
Example outputs demonstrating the integration of roadDB with auxiliary packages. Example results through roadDB with database snapshot v. 2026-09-18. a) Simple geographic plot with 2,127 localities obtained through code presented in section 3.4.2 using ‘sf’ and ‘tmap’; Background map courtesy of OpenStreetMap Contributors, published under Open Database License. b) Aoristic sum of the temporal distribution of 29,087 assemblages dating between 3,000,000 and 20,000 years BP, obtained through code shown in section 3.4.3 using ‘aoristAAR’. c) Relative abundance of assemblage categories over time. No permits for human remains were required for the described study, which complied with all relevant regulations. d) Time series of annual mean temperature and min/max temperature ranges of Hohle Fels Cave during the Late Pleistocene and Holocene, based on data from Krapp et al. [61] using ‘pastclim’ and ‘ggplot2’. e) Radiocarbon calibration of the Aurignacian “flute” layer AH Vb at Hohle Fels [62] using ‘rcarbon’. Reproducible code for all figures is available in the supplementary S1 File. Package logos reprinted from ‘tmap’, ‘aoristAAR’, ‘pastclim’ and ‘rcarbon’ under a CC BY license, with permission from Dr. Martijn Tennekes, Dr. Nils Müller-Scheeßel, Dr. Michaela Leonardi and Prof. Enrico R. Crema, original copyright 2026.

#### 3.4.1 Installation.

RoadDB can be installed directly from the Comprehensive R Archive Network (CRAN) or from Github.

# CRAN installation (recommended)

install.packages("roadDB")

# Github installation (Development version)

install.packages("devtools")

devtools::install_github("sommergeo/roadDB")

#### 3.4.2 Mapping.

R offers a wide variety of tools for processing and displaying geographical data in maps. Here we use the package ‘sf’ (v. 1.1−2 [58],) to transform the roadDB outputs into simple features, a spatial vector format, and visualize them with the ‘tmap’ package (v. 4.4−1 [59],). The resulting map is displayed in Fig 3a.

# Load libraries

library(roadDB)  # this package

library(sf)     # for spatial objects

library(tmap)   # for mapping

# Use coordinate columns to create a spatial dataset

sites < - road_get_localities() |>

      st_as_sf(coords = c("coord_x", "coord_y"),

      crs = 4326) # WGS84 decimal coordinate system

# Display map

tm_shape(sites) +

  tm_basemap("OpenStreetMap") +

  tm_dots()

#### 3.4.3 Aoristic analysis.

Aoristic analysis is a method used to distribute events with uncertain dates probabilistically across their possible time ranges, rather than assigning them to a single fixed point in time. Here we demonstrate how a timeline of aoristic sums can be calculated from roadDB outputs with the package ‘aoristAAR’ (v. 0.1.0 [60],). The resulting graph is displayed in Fig 3b.

library(aoristAAR)

assemblages < - road_get_assemblages()

aoristic_sum <- aorist(assemblages, from="age_min", to="age_max",

         stepwidth = 20000)

plot(aoristic_sum, type = "l")

### 3.5 Using roadDB responsibly

Since the ROAD database is constantly evolving and the roadDB snapshot is updated regularly, we recommend saving a local copy of the queried tables. This ensures reproducibility, even if a later snapshot yields different results. Furthermore, we recommend validating the data retrieved from roadDB, for example by consulting the original literature (available through *road_get_publications()*) and searching for newer publications not yet included in ROAD. Note that ROAD is curated by experienced researchers and trained research assistants at ROCEEH, but may still contain errors. The information is recorded to closely follow the interpretations of the original literature and does not reflect the opinions of the curators. Nevertheless, some subjective choices had to be made, as the database is sourced from over 150 years of literature in diverse languages and research traditions, which naturally introduces a degree of ambiguity.

This package provides simplified access to the most frequently used contents in ROAD. Users with more specific or complex requests can obtain full read access using *road_get_sql()* or via the browser interface (https://www.roceeh.uni-tuebingen.de/roadweb/smarty_road_simple_search.php). In either case, users should familiarize themselves with the database structure (https://doi.org/10.5281/zenodo.17720874) and the detailed table descriptions (https://doi.org/10.5281/zenodo.17660207).

## 4. Discussion

The scientific value of roadDB lies not only in its disciplinary breadth, but also in its suitability for macroscale inference. As argued by Perreault [1], archaeology is particularly well positioned to identify large-scale patterns that emerge only through the aggregation of data across vast spans of time and space, a research strategy often described as macroarchaeology. roadDB directly supports this agenda by enabling the rapid assembly of continental and intercontinental-scale datasets, the systematic compilation of cultural, biological, and environmental variables, and the integration of ROAD data with external climatic, chronological, and spatial models.

### 4.1 roadDB in the R ecosystem

roadDB functions as a structured data provider within a growing ecosystem of R packages dedicated to human evolutionary and paleoenvironmental research. It supplies synthesized archaeological, anthropological, paleoenvironmental and spatio-temporal information from archaeological sites. It thus complements specialized packages providing data from chronometric dating (‘p3k14c’ [63], ‘c14bazAAR’ [64]), paleontology (‘neotoma2’ [65], ‘rgbif’ [66], ‘paleobioDB’ [67]), paleoclimate reconstruction (‘pastclim’ [68], ‘rpaleoclim’ [69]) and geology (‘rmacrostrat’ [70]).

By providing structured access to ROAD, it complements input to tools focused on specific analytical domains, including ecological niche modeling (‘ppgm’ [71], ‘tidysdm’ [72]), age modeling (‘BayLum’ [73], ‘rcarbon’ [74], ‘coffee’ [75], ‘oxcAAR’ [76]), time series analysis (‘baorista’ [77], ‘deeptime’ [78], ‘aion’ [79], ‘datplot’ [80], ‘ArchaeoPhases’ [81]), archaeological analysis (‘seriation’ [82], ‘tabula’ [83], ‘tesselle’ [84]), paleontological analysis (‘analogue’ [85], ‘paleotree’ [86], ‘zooarch’ [87]) paleoenvironmental reconstruction (’crestr’ [88],), and spatial analysis (e.g., [58]).

Crucially, roadDB does not attempt to replace these tools. Rather, it acts as an infrastructure layer, ensuring that ROAD can be integrated directly into reproducible analytical workflows. In this sense, roadDB is best understood not as a standalone analytical tool, but as a connector package that embeds ROAD within a growing ecosystem of computational methods for research on human evolution. As archaeological and paleoenvironmental research increasingly shifts toward large-scale synthesis, comparative analysis, and explicit modeling, tools like roadDB become essential for transforming curated legacy datasets into engines of new discovery.

### 4.2 Openness, reproducibility, and FAIR principles

Open science has become a central concern across archaeology, particularly as research increasingly relies on large datasets and computational methods. Marwick and colleagues [5] argue that traditional archaeological publications often prioritize final results over the analytical processes that produced them and thereby leave key decisions about data selection, transformation and modeling implicit or undocumented. In computational research, this lack of transparency substantially limits reproducibility as even minor undocumented choices can strongly influence results. From this perspective, reproducibility is not a binary property but a spectrum ranging from studies that present only narrative interpretations and figures to those that provide data, code, and fully executable analytical workflows. Moving archaeological research along this spectrum requires not only open access to publications and data, but also open methods: scripted, inspectable, and reusable analytical procedures implemented in transparent software environments. Programmatic access to data plays a key role in this transition. While many archaeological datasets are now openly available, they are often accessed through manual downloads, interactive web interfaces, or undocumented filtering steps. Such workflows hinder reproducibility by making it difficult to reconstruct exactly which data were used and how they were selected. Here a shift from data ownership to data stewardship, in which researchers treat datasets as shared community resources and document their analytical use in ways that facilitate verification, reuse, and extension is called for [4,5].

The roadDB package directly supports this approach by embedding access to ROAD within a scripted R environment. Queries to ROAD become explicit, shareable code, rather than undocumented actions carried out through a graphical interface. This makes data selection and filtering part of the analytical record and allows workflows to be rerun, modified, and audited. By operating within R, an open-source environment widely used for reproducible research, roadDB aligns with best practices for transparent computational analysis. In addition, roadDB supports the FAIR data principles (Findable, Accessible, Interoperable, Reusable). ROAD records are retrieved through structured queries, improving findability and accessibility. Outputs are returned as tidy data frames, enhancing interoperability with other analytical tools in the R ecosystem. Reusability is supported by clear documentation of function behavior, argument values, and database versions, enabling researchers to specify precisely which data release underpins a given analysis. By lowering the barrier between a complex relational database and reproducible analytical workflows, roadDB provides another way of using ROAD as an active component of open archaeological science. In doing so it provides a concrete implementation of the open methods agenda, demonstrating how curated macroarchaeological databases can be integrated into transparent, repeatable, and extensible research pipelines.

## 5. Conclusion

roadDB is a practical R package that enables the integration of open, structured, and synthesized data from the ROCEEH Out of Africa Database (ROAD) into the R ecosystem. Its optimal compatibility with auxiliary R packages makes it a valuable data provider for further scientific analysis in prehistoric and paleoenvironmental research. By facilitating transparent data access and reusable workflows, roadDB constitutes a cornerstone for open and reproducible science.

## Supporting information

S1 File Reproducible code for the examples displayed in Fig 3. (ZIP)
