# Supplementary material for: roadDB: An R package for exploring early human prehistory and environments with the ROCEEH Out of Africa Database
Source: PLoS One. 2026 Oct 7;21(10):e0359952. doi: 10.1371/journal.pone.0359952 (PMC13645032; doi:10.1371/journal.pone.0359952)
Supplement: Supplementary file 2 [file pone.0359952.s002.docx]

# Editor:

Thank you for your patience during the review of this manuscript, and I apologize for this extended review. Despite repeated efforts, we were unable to secure an additional external review. Rather than delay the manuscript further, I have evaluated it on the basis of the review received together with my own editorial assessment. Both the reviewer and I were able to install and test the package successfully and evaluated the manuscript very favorably, and I think it is close to being suitable for publication in PLOS ONE. I am therefore requesting a minor revision. Provided these targeted clarifications are addressed, I do not anticipate that further external review will be necessary.

Response: Thank you very much for your efforts and for taking the initiative to review our manuscript yourself when no additional reviewers could be found. We know how difficult it has become to find reviewers. We are truly grateful for your dedication to ensuring a fair and timely evaluation.

We are very happy that our manuscript has been well received in general, and we are confident that the current edits we have made make the open issues much clearer. Additionally, we allowed ourselves to extend the manuscript with one additional function (road_get_sql) that was released with version 0.2 of the package.

My main question focuses on the “snapshot” capability of the package. The text states “This versioning ensures that identical queries executed at different time points yield consistent results,” but the package startup message says “results may vary over time.” It is not clear from the manuscript or package documentation whether previous snapshots of the database remain publicly accessible and, if so, whether users can select a particular snapshot through roadDB. If prior snapshots are available, this would be a helpful addition to the package API (if feasible). If not, then the language in the manuscript should be updated to clarify this.

Response: The reproducibility across different snapshots is indeed a critical point. We considered running multiple instances, one for each snapshot, but servers will eventually shut down. This would only shift the problem into the future. We therefore decided to work on an extension of the package that allows users to download a copy of the snapshot in the SQLite format. This format works serverless and self-contained on local hardware and does not require users to install and run a full-fledged database management system, as would be required if we distributed the database as-is in PostgreSQL (like our annual database dump at https://doi.org/10.5281/zenodo.18171454). While this removes many hurdles for users, we have to translate the entire package, as the SQL dialects of PostgreSQL and SQLite are incompatible. We are currently developing this in a dedicated branch, but it will probably not be merged until next year. Unfortunately, there are still too many open decisions at the moment to include this extension in the manuscript.

We have therefore edited sections 2.1 and 3.5 and the caption of figure 1 as follows to address the problem and suggest solutions:

Lines 148–149: “Note that query results may differ each time the roadDB database is updated.”

Lines 162–165: “This versioning ensures that identical queries executed at different time points yield consistent results, as long as the snapshot is active. The snapshot is updated every three months. Consequently, the approach supports reproducibility of analyses and enhances transparency in scientific reporting, e.g. by stating the snapshot version date.”

Lines 358–374: “Since the ROAD database is constantly evolving and the roadDB snapshot is updated regularly, we recommend saving a local copy of the queried tables. This ensures reproducibility, even if a later snapshot yields different results.”

Also, the package documentation has a great section on “Using roadDB responsibly”. Bringing a few ideas from this section into the main manuscript, particularly a brief discussion of the database’s coverage and interpretation, and the packages scope relative to the entirety of the underlying database available through ROAD, would be very helpful for prospective users of the package.

Response: Thank you, we added the section “3.5 Using roadDB responsibly” to cover these points:

Lines 359–374: “Since the ROAD database is constantly evolving and the roadDB snapshot is updated regularly, we recommend saving a local copy of the queried tables. This ensures reproducibility, even if a later snapshot yields different results. Furthermore, we recommend validating the data retrieved from roadDB, for example by consulting the original literature (available through road_get_publications()) and searching for newer publications not yet included in ROAD. Note that ROAD is curated by experienced researchers and trained research assistants at ROCEEH, but may still contain errors. The information is recorded to closely follow the interpretations of the original literature and does not reflect the opinions of the curators. Nevertheless, some subjective choices had to be made, as the database is sourced from over 150 years of literature in diverse languages and research traditions, which naturally introduces a degree of ambiguity.

This package provides simplified access to the most frequently used content in ROAD. Users with more specific or complex requests can obtain full read access using road_get_sql() or via the browser interface (https://www.roceeh.uni-tuebingen.de/roadweb/smarty_road_simple_search.php). In either case, users should familiarize themselves with the database structure (https://doi.org/10.5281/zenodo.17720874) and the detailed table descriptions (https://doi.org/10.5281/zenodo.17660207).”

Finally, please address the reviewer’s suggestion to make the figure captions more informative and self-contained, and some correct minor inconsistencies in terminology, numbering, citations, and software versions. A few specific typos I noticed are:

- “3.3 Helper functions” and “3.3 Examples” are both numbered 3.3. Response: corrected

- p3k13c appears to be a typo for p3k14c. Response: indeed, corrected

- The citation associated with the tidyverse appears to be incorrect; reference 57 is assertthat. Response: corrected

- Rpostgres should be RPostgres. Response: corrected

- teselle should be tesselle. Response: corrected

- “University of Chicage Press” should be “University of Chicago Press.” Response: corrected

- There is a doubled parenthesis around the citation “((4,5))” in the Discussion. Response: corrected

# Reviewer:

Reviewer #1: This paper presents a useful R Package for directly accessing the ROCEEH database, without users needing to do manual SQL queries on the project website. Overall the manuscript is clearly written, and the three figures are informative—especially the workflow in Figure 1 and the function and arguments table given in Figure 2. My only comment to consider is that the figure captions are pretty thin—since space is not a constraint here, I would suggest making the captions more informative.

I also was able to download the package and test the various functions—all worked perfectly fine, sometimes queries took a bit long to run but that is understandable given the size of the datasets. ROCEEH provide incredible data for the last 3 Myr of hominin evolution I would anticipate this package being widely used.

Response: Thank you very much for your positive and constructive feedback on our manuscript. We greatly appreciate your time and effort in reviewing our work, testing the package, and providing such valuable comments.

We are pleased to hear that you found the package useful and the manuscript clearly written. Following your suggestion, we have extended the figure captions accordingly to make them more informative. We hope that the package will indeed be useful to many.

# Journal Requirements:

When submitting your revision, we need you to address these additional requirements.

1. Please ensure that your manuscript meets PLOS ONE's style requirements, including those for file naming. The PLOS ONE style templates can be found at

https://journals.plos.org/plosone/s/file?id=wjVg/PLOSOne_formatting_sample_main_body.pdf and

https://journals.plos.org/plosone/s/file?id=ba62/PLOSOne_formatting_sample_title_authors_affiliations.pdf

We deleted the “Author contribution” and “Data availability” statement, if that is what was not compliant with the template. If something else was the problem, please provide more specific instructions.

2. In your manuscript, please provide additional information regarding the specimens used in your study. Ensure that you have reported human remain specimen numbers and complete repository information, including museum name and geographic location.

If permits were required, please ensure that you have provided details for all permits that were obtained, including the full name of the issuing authority, and add the following statement:

'All necessary permits were obtained for the described study, which complied with all relevant regulations.'

If no permits were required, please include the following statement:

'No permits were required for the described study, which complied with all relevant regulations.'

For more information on PLOS One's requirements for paleontology and archeology research, see https://journals.plos.org/plosone/s/submission-guidelines#loc-paleontology-and-archaeology-research.

These instructions are unclear. Do we need to add a dedicated “Ethics section”? Such a section is nowhere described in the formatting instructions. Also, it is clearly described that this is a database study, that does not directly rely on any human or archaeological remains, but literature. We added the sentence “No permits for human remains were required for the described study, which complied with all relevant regulations.” to figure 3 captions, where “human remains” appear as a category in figure 3c).

3. Please note that PLOS One has specific guidelines on code sharing for submissions in which author-generated code underpins the findings in the manuscript. In these cases, we expect all author-generated code to be made available without restrictions upon publication of the work. Please review our guidelines at https://journals.plos.org/plosone/s/materials-and-software-sharing#loc-sharing-code and ensure that your code is shared in a way that follows best practice and facilitates reproducibility and reuse.

This request is unclear, because all code is attached as supporting information S1 File and referenced in figure 3 captions. What should be changed?

4. We note that the grant information you provided in the ‘Funding Information’ and ‘Financial Disclosure’ sections do not match.

When you resubmit, please ensure that you provide the correct grant numbers for the awards you received for your study in the ‘Funding Information’ section.

We updated the funding information with a request to change the online form in the cover letter.

5. Thank you for stating the following financial disclosure:

“This work was funded by the Heidelberg Academy of Sciences and Humanties (https://en.hadw-bw.de/). We acknowledge support by the Open Access Publishing Fund of the University of Tübingen (https://uni-tuebingen.de/en/216529).”

Please state what role the funders took in the study. If the funders had no role, please state: "The funders had no role in study design, data collection and analysis, decision to publish, or preparation of the manuscript."

If this statement is not correct you must amend it as needed.

Please include this amended Role of Funder statement in your cover letter; we will change the online submission form on your behalf.

We added the role of the funders to the funding information with a request to change the online form in the cover letter.

6. Thank you for stating the following in the Acknowledgments Section of your manuscript:

“This work was funded by the Heidelberg Academy of Sciences. The research center “The Role of Culture in Early Expansions of Humans” (ROCEEH) and colleagues in many countries contributed to ROAD. Most important of all, we are grateful to the dozens of student research assistants at the Universities of Frankfurt and Tübingen who have entered data into ROAD since 2009. Without their hard work and perseverance, as well as their competence in languages we cannot understand, ROAD and consequently this R package could never have taken its current shape. We acknowledge support by the Open Access Publishing Fund of the University of Tübingen.”

We note that you have provided funding information that is not currently declared in your Funding Statement. However, funding information should not appear in the Acknowledgments section or other areas of your manuscript. We will only publish funding information present in the Funding Statement section of the online submission form.

Please remove any funding-related text from the manuscript and let us know how you would like to update your Funding Statement. Currently, your Funding Statement reads as follows:

“This work was funded by the Heidelberg Academy of Sciences and Humanties (https://en.hadw-bw.de/). We acknowledge support by the Open Access Publishing Fund of the University of Tübingen (https://uni-tuebingen.de/en/216529).”

Please include your amended statements within your cover letter; we will change the online submission form on your behalf.

We removed the funding information from acknowledgements.

7. We note that Figure 3 in your submission contain map images which may be copyrighted. All PLOS content is published under the Creative Commons Attribution License (CC BY 4.0), which means that the manuscript, images, and Supporting Information files will be freely available online, and any third party is permitted to access, download, copy, distribute, and use these materials in any way, even commercially, with proper attribution. For these reasons, we cannot publish previously copyrighted maps or satellite images created using proprietary data, such as Google software (Google Maps, Street View, and Earth). For more information, see our copyright guidelines: http://journals.plos.org/plosone/s/licenses-and-copyright.

We require you to either (1) present written permission from the copyright holder to publish these figures specifically under the CC BY 4.0 license, or (2) remove the figures from your submission:

1. You may seek permission from the original copyright holder of Figure 3 to publish the content specifically under the CC BY 4.0 license.

We recommend that you contact the original copyright holder with the Content Permission Form (http://journals.plos.org/plosone/s/file?id=7c09/content-permission-form.pdf) and the following text:

“I request permission for the open-access journal PLOS ONE to publish XXX under the Creative Commons Attribution License (CCAL) CC BY 4.0 (http://creativecommons.org/licenses/by/4.0/). Please be aware that this license allows unrestricted use and distribution, even commercially, by third parties. Please reply and provide explicit written permission to publish XXX under a CC BY license and complete the attached form.”

Please upload the completed Content Permission Form or other proof of granted permissions as an ""Other"" file with your submission.

In the figure caption of the copyrighted figure, please include the following text: “Reprinted from [ref] under a CC BY license, with permission from [name of publisher], original copyright [original copyright year].”

2. If you are unable to obtain permission from the original copyright holder to publish these figures under the CC BY 4.0 license or if the copyright holder’s requirements are incompatible with the CC BY 4.0 license, please either i) remove the figure or ii) supply a replacement figure that complies with the CC BY 4.0 license. Please check copyright information on all replacement figures and update the figure caption with source information. If applicable, please specify in the figure caption text when a figure is similar but not identical to the original image and is therefore for illustrative purposes only.

The following resources for replacing copyrighted map figures may be helpful:

USGS National Map Viewer (public domain): http://viewer.nationalmap.gov/viewer/

The Gateway to Astronaut Photography of Earth (public domain): http://eol.jsc.nasa.gov/sseop/clickmap/

Maps at the CIA (public domain): https://www.cia.gov/library/publications/the-world-factbook/index.html and https://www.cia.gov/library/publications/cia-maps-publications/index.html

NASA Earth Observatory (public domain): http://earthobservatory.nasa.gov/

Landsat: http://landsat.visibleearth.nasa.gov/

USGS EROS (Earth Resources Observatory and Science (EROS) Center) (public domain): http://eros.usgs.gov/#

Natural Earth (public domain): <http://www.naturalearthdata.com/>

Response:

Figure 3a uses the OpenStreetMap as basemap, which uses the Open Database License. According to https://www.openstreetmap.org/copyright : “Where you use OpenStreetMap data, you are required to do the following two things:
1. Provide credit to OpenStreetMap by displaying our attribution notice.
2. Make clear that the data is available under the Open Database License.”

The figure caption was updated accordingly (line 344–345): “Background map courtesy of OpenStreetMap Contributors, published under Open Database License.”

We added credit for the hex logos to the captions where owner permissions were received, uploaded the signed forms, and removed the others. “Package logos reprinted from ‘tmap’, ‘aoristAAR’, ‘pastclim’ and ‘rcarbon’ under a CC BY license, with permission from Dr. Martijn Tennekes, Dr. Nils Müller-Scheeßel, Dr. Michaela Leonardi and Prof. Enrico R. Crema, original copyright 2026.” (lines 353–356)

8. We note that Figure 1 in your submission contain copyrighted images. All PLOS content is published under the Creative Commons Attribution License (CC BY 4.0), which means that the manuscript, images, and Supporting Information files will be freely available online, and any third party is permitted to access, download, copy, distribute, and use these materials in any way, even commercially, with proper attribution. For more information, see our copyright guidelines: http://journals.plos.org/plosone/s/licenses-and-copyright.

We require you to either (1) present written permission from the copyright holder to publish these figures specifically under the CC BY 4.0 license, or (2) remove the figures from your submission:

1. You may seek permission from the original copyright holder of Figure 1 to publish the content specifically under the CC BY 4.0 license.

We recommend that you contact the original copyright holder with the Content Permission Form (http://journals.plos.org/plosone/s/file?id=7c09/content-permission-form.pdf) and the following text:

“I request permission for the open-access journal PLOS ONE to publish XXX under the Creative Commons Attribution License (CCAL) CC BY 4.0 (http://creativecommons.org/licenses/by/4.0/). Please be aware that this license allows unrestricted use and distribution, even commercially, by third parties. Please reply and provide explicit written permission to publish XXX under a CC BY license and complete the attached form.”

Please upload the completed Content Permission Form or other proof of granted permissions as an "Other" file with your submission.

In the figure caption of the copyrighted figure, please include the following text: “Reprinted from [ref] under a CC BY license, with permission from [name of publisher], original copyright [original copyright year].”

2. If you are unable to obtain permission from the original copyright holder to publish these figures under the CC BY 4.0 license or if the copyright holder’s requirements are incompatible with the CC BY 4.0 license, please either i) remove the figure or ii) supply a replacement figure that complies with the CC BY 4.0 license. Please check copyright information on all replacement figures and update the figure caption with source information. If applicable, please specify in the figure caption text when a figure is similar but not identical to the original image and is therefore for illustrative purposes only.

Response: We replaced the R logo (CC BY SA) with the letter “R” (ass suggested in case 2i).

9. We are unable to open your Supporting Information file [S1_file.R]. Please kindly revise as necessary and re-upload.

We reuploaded the file. It is a standard R project file, and can be opened with a current version of R.

10. If the reviewer comments include a recommendation to cite specific previously published works, please review and evaluate these publications to determine whether they are relevant and should be cited. There is no requirement to cite these works unless the editor has indicated otherwise.

Response: No additional references were recommended. Our mistake of citing `assertthat` instead of `tidyverse` was corrected.

11. Please review your reference list to ensure that it is complete and correct. If you have cited papers that have been retracted, please include the rationale for doing so in the manuscript text, or remove these references and replace them with relevant current references. Any changes to the reference list should be mentioned in the rebuttal letter that accompanies your revised manuscript. If you need to cite a retracted article, indicate the article’s retracted status in the References list and also include a citation and full reference for the retraction notice.

Response: All references have been reviewed.
